# Dynamic Proteomic Changes in Tumor and Immune Organs Reveal Systemic Immune Response to Tumor Development

**DOI:** 10.1016/j.mcpro.2024.100756

**Published:** 2024-03-28

**Authors:** Zhike Li, Shuwen Liu, Zhouyong Gao, Linlin Ji, Jiaqi Jiao, Nairen Zheng, Xianju Li, Guangshun Wang, Jun Qin, Yi Wang

**Affiliations:** 1 State Key Laboratory of Medical Proteomics, Beijing Proteome Research Center, National Center for Protein Sciences (Beijing), Beijing Institute of Lifeomics, Beijing, China; 2 Department of Thoracic Surgery, Baodi Clinical College, Tianjin Medical University, Tianjin, China; 3 Department of Child Health Care, Kunshan Maternity and Child Health Care Institute, Kunshan, China; 4 Department of Thoracic Surgery, Weifang People’s Hospital, Weifang, China

**Keywords:** host’s macroenvironment, tumor development, immune organs, MFC allograft mouse model, temporal proteomic atlas, MEK, DDR2

## Abstract

In orthotopic mouse tumor models, tumor progression is a complex process, involving interactions among tumor cells, host cell-derived stromal cells, and immune cells. Much attention has been focused on the tumor and its tumor microenvironment, while the host’s macroenvironment including immune organs in response to tumorigenesis is poorly understood. Here, we report a temporal proteomic analysis on a subcutaneous tumor and three immune organs (LN, MLN, and spleen) collected on Days 0, 3, 7, 10, 14, and 21 after inoculation of mouse forestomach cancer cells in a syngeneic mouse model. Bioinformatics analysis identified key biological processes during distinct tumor development phases, including an initial acute immune response, the attack by the host immune system, followed by the adaptive immune activation, and the build-up of extracellular matrix. Proteomic changes in LN and spleen largely recapitulated the dynamics of the immune response in the tumor, consistent with an acute defense response on D3, adaptive immune response on D10, and immune evasion by D21. In contrast, the immune response in MLN showed a gradual and sustained activation, suggesting a delayed response from a distal immune organ. Combined analyses of tumors and host immune organs allowed the identification of potential therapeutic targets. A proof-of-concept experiment demonstrated that significant growth reduction can be achieved by dual inhibition of MEK and DDR2. Together, our temporal proteomic dataset of tumors and immune organs provides a useful resource for understanding the interaction between tumors and the immune system and has the potential for identifying new therapeutic targets for cancer treatment.

## Highlights

• A temporal proteomic atlas of tumor and three immune organs in an MFC allograft model.
• Tumor induces acute response, then adaptive immunity, ECMs buildup, and immune evasion.
• Proteomic changes in LN and spleen echoed the dynamic immune response in the tumor.
• Identified and validated DDR2 and MEK as potential therapeutic targets.

## Graphical Abstract

**Figure:**
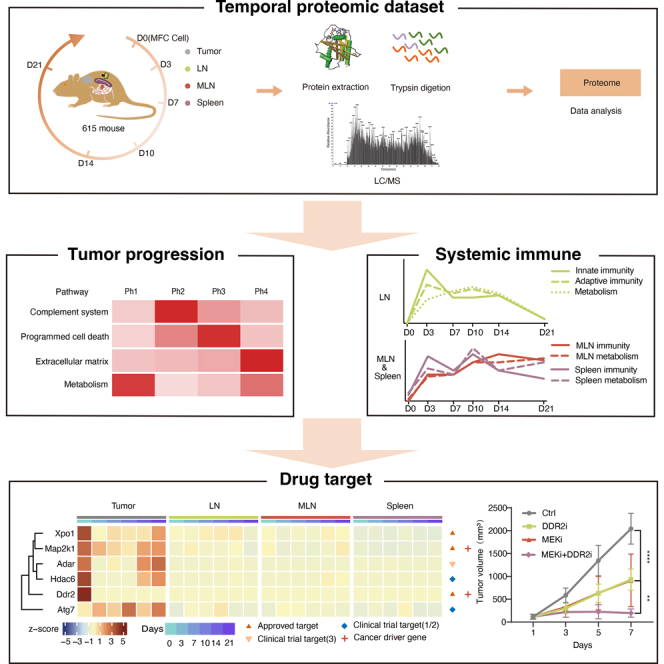

## In Brief

This study presented a temporal proteomic analysis on a subcutaneous tumor and immune organs during tumor implantation. The tumors encountered an acute immune response, followed by adaptive immune activation, extracellular matrix build-up, and immune evasion. Proteomic changes in lymph nodes and spleen recapitulated the dynamic immune response in the tumor, while the mesenteric lymph nodes showed progressive and sustained activation, suggesting a delayed response from a distal immune organ. Differentially overexpressed protein analysis identified and validated potential therapeutic targets.

Upper gastrointestinal cancer is one of the most common malignancies worldwide, with gastric cancer (GC) and esophageal cancer (EC) ranking fifth and seventh for incidence and fourth and sixth for mortality, respectively (1). Asia experiences the most significant occurrence and mortality rates, accounting for over 70% of new cases and deaths worldwide (1). Most upper gastrointestinal tumors are diagnosed at an advanced stage and pose a significant challenge to the healthcare system due to their aggressive manifestations. Early-stage esophageal and gastric cancers are commonly treated with endoscopic resection, while advanced-stage cases typically require surgery after undergoing neoadjuvant chemoradiotherapy (NCRT) or radical chemoradiotherapy (2). Despite these interventions, the 5-year survival rate remains below 50% due to the high likelihood of distant recurrence.

Molecularly driven treatment approaches targeting specific oncogenic events or pathways have become mainstream and are first-line treatment options in lung cancer (3), melanoma (4), and metastatic colorectal cancer (5). Therapy targets in EC include oncogenes (EGFR, HER-2, VEGFR, c-Met, etc.) and immune checkpoints (PD-1/PDL-1) (6). The only approved target in GC is HER-2. However, these therapies have not resulted in substantial improvements in survival, in part because actionable genetic mutations occur only in a small population of EC and GC. There is an urgent need to develop single-agent or combination therapies in clinical practice.

The tumorigenesis and progression are complex processes involving interactions among tumor cells, stromal cells, and the immune defense system of the body. Studies from the past few decades suggest that the immune system plays a critical role in maintaining the balance between immune recognition and tumor development, with a dual capacity to promote and suppress tumor growth (7). At present, the understanding of the relationship between tumor and the immune system mostly revolves around the role of different immune cells in the tumor microenvironment, but the changes in the host's immune macroenvironment in response to tumorigenesis are poorly understood (8, 9). A recent study characterized the systemic immune landscape across eight commonly used mouse tumor models through immune cell changes by mass cytometry. Their results showed that tumor-driven dynamic changes in immune cell organization and function throughout the organism, culminating in diminished response to orthogonal immune challenges, and tumor resection was sufficient to restore systemic immune status (10).

Patient-derived tissues (PDX) model plays a pivotal role in preclinical drug testing and optimization in cytotoxic cancer drug discovery (11). However, the inability to replicate the potential tumor-promoting and anti-tumor impacts of stromal cells and immune cells renders them inappropriate for the targeted treatment of tumor microenvironment (TME) component (12). By employing synergic tumor models, one can gain a deeper insight into the ramifications of immunity on the development of tumors. Mouse forestomach carcinoma (MFC) is a cell line with a strong tendency for lung metathesis (13). Due to the structural similarity between the mouse forestomach and the human esophagus, MFC has been used as a model for both EC and GC (14). In this study, we used MFC derived allograft mouse model to characterize dynamic proteomic changes of subcutaneous tumors and three immune organs (draining lymph node, mesenteric lymph node, and spleen) at different time points after implantation. Bioinformatics analyses were used to identify key pathways and molecular processes occurring in both tumor and immune microenvironment. Furthermore, we tested the feasibility of using proteomic profiling to identify potential therapeutic targets and validated the approach in the MFC model.

## Experimental Procedures

### Experimental Design and Statistical Rationale

A total of 92 samples for tumors, LN, MLN, and spleen on days 0, 3, 7, 10, 14, and 21 after MFC cell inoculation in a syngeneic mouse model were measured using LC-MS. The tissue-specific proteins identified by the Venn diagram and WGCNA and Functional enrichment analyses were performed using Metascape (https://metascape.org/). Significantly enriched proteins in the MFC tumor (D3 *versus* D0) were identified using a two-sided Student’s *t* test with a 10-fold change and Benjamini-Hochberg correction at a *p*-value threshold <0.05. Co-expression analysis using the fuzzy c-means (FCM) clustering algorithm disclosed seven protein clusters within the MFC tumor. The comparison of D3/D7/D10/D14/D21 with D0 individually delineated the variation in proteins, forming the basis for inferring the functional implications within LN, MLN, and spleen. Through integrated analyses encompassing tumors and host immune organs, a total of 110 drug targets were identified within the DrugBank.

### Cell and Reagents

MFC cell line was obtained from Peking Union Medical College. MFC cells were cultured in RPMI-1640 medium supplemented with 10% FBS (Gibco), and 1% penicillin/streptomycin (Gibco). Trametinib (GSK1120212; Cat. No. M1759) and sitravatinib (Cat. No. M9626) were purchased from Abmole.

### Animals for Experiments

All animal experiments were approved by the animal care regulations of the Institutional Animal Care and Use Committee of the National Protein Science Center (Beijing Proteome Research Center) (IACUC-20210702-26MT). Eight-week-old male 615 mice were purchased from the Institute of Laboratory Animals Science, CAMS & PUMC and housed under a standard SPF (specific pathogen-free) laboratory environment. Six-week-old male BALB/c nude mice were purchased from Beijing Vital River Laboratory Animal Technology Co, Ltd, Beijing, China.

The “615” strain was created through the hybridization of albino female mice from the Kunming strain and black male mice from the C57BL inbred strain, imported from the former Soviet Union. Subsequent sibling mating for over 20 generations resulted in the establishment of a brown-colored mouse strain, named “615” based on the month and year of its inception (15). The immune competent “615” was used to investigate the temporal proteomic change in tumor and various immune organs.

### *In Vivo* Cell-Derived Allograft Experiments

For cell-derived allograft experiments, Eight-week-old male immune-competent 615 mice were used. MFC cells (2 × 10^6^ cells) were subcutaneously implanted in the right flank of mice. Tumor and three immune organs (LN, MLN, and spleen) were collected at 6 time points: D0, D3, D7, D10, D14, and D21. Each time point had at least three biological replicates for temporal proteomic analysis.

### Protein Extraction and Trypsin Digestion

Samples were subjected to protein extraction in lysis buffer (1% sodium deoxycholate, 10 mM Tris(2-carboxyethyl) phosphine, 40 mM 2-chloroacetamide, and 100 mM Tris–HCl pH8.8). After heating at 95 °C for 5 min and sonicating for 5 min (3 s on and 3 s off, amplitude 25%), the tissue lysates were centrifuged at 16,000*g* for 10 min at 4 °C, and the supernatants were collected as whole tissue extract (WTE). 100 g protein (the protein concentration determined by Thermo Nanodrop One) was digested overnight with trypsin (Promega) at 37 °C, and the digestion was stopped by formic acid at the final concentration of 1%. Precipitated sodium deoxycholate was removed by centrifugation at 4 °C with 16,000*g* for 10 min. The supernatants were collected, desalted, vacuum-dried and stored at −80 °C until subsequent liquid chromatography-tandem mass spectrometry (LC-MS/MS) analysis.

### LC-MS/MS Analysis

Dried peptide samples were re-dissolved in Solvent A (0.1% formic acid in water) and loaded to a trap column (100 μm × 2 cm, homemade; particle size, 1.9 μm; pore size, 120 Å; SunChrom) with a max pressure of 280 bar using Solvent A, then separated on a home-made 150 μm × 30 cm silica microcolumn (particle size, 1.9 μm; pore size, 120 Å; SunChrom) with a gradient of 5 to 35% mobile phase B (80% acetonitrile and 0.1% formic acid) at a flow rate of 600 nl/min for 140 min and washed for 10 min using 95% mobile phase B (0.1% formic acid in acetonitrile).

The eluted peptides were ionized under 2 kV. MS was operated under a data-dependent acquisition (DDA) mode. For detection with a Fusion Lumos mass spectrometer, a precursor scan was carried out in the Orbitrap by scanning m/z 300 to 1400 with a resolution of 120,000 at 200 m/z. The most intense ions selected under top-speed mode were isolated in Quadrupole with a 1.6 m/z window and fragmented by higher energy collisional dissociation (HCD) with a normalized collision energy of 35%, then measured in the linear ion trap using the rapid ion trap scan rate. Automatic gain control targets were 5 × 10 e^5^ ions with a max injection time of 50 ms for full scans and 5 × 10 e^3^ with 35 ms for MS/MS scans. Dynamic exclusion time was set as 18 s.

### Protein Identification and Quantification

MS raw files were processed with the Z-system proteomics workstation (http://103.79.27.115:9000/). MS raw files were searched against the National Center for Biotechnology Information (NCBI) Ref-seq mouse proteome database (updated on 04/07/2013, 27,414 entries) in the Mascot search engine (version 2.3, Matrix Science Inc). The mass tolerances were 20 ppm for precursor ions and 0.5 Da for product ions. The minimal peptide length was seven amino acid long. Cysteine carbamidomethylation was set as a fixed modification, and protein N-acetylation and oxidation of methionine were considered variable modifications. 1% FDR on the peptide estimated by searching a decoy database was allowed. Proteins were quantified by a label-free, intensity-based absolute quantification (iBAQ) approach (16) and further normalized into iFOT (intensity-based the fraction of total multiplied by 10^5^). To mitigate the issue of high dimensionality caused by the potential encoding of multiple protein isoforms or variants by a single gene, the proteins were collapsed into gene-level.

### Proteome Data Filtering

The proteomic data was filtered to three datasets at different levels according to the following criteria (supplemental Fig. S1*A*): (1) DataSet 1 included all 12,160 identified GeneProducts (GPs) on 1% peptide FDR; (2) DataSet 2 included 7950 proteins that were required to have at least one unique peptide & two strict peptides or three strict peptides, or one unique strict peptide with ion score greater than or equal to 40; and (3) DataSet 3 included 6401 proteins that were identified in at least two of three replicates at one timepoint.

“Unique peptide” refers to a specific peptide sequence that is exclusively found within a particular protein sequence. “Strict peptide” refers to a peptide identified in mass spectrometry analysis with an ion score of 20 or higher.

### Weighted Gene Correlation Network Analysis

The co-expression network analysis was specifically performed for 92 samples using R package Weighted Gene Correlation Network Analysis (WGCNA; version 1.72-1) (17). We used iFOT in DataSet 3 to construct the expression matrix. First, the sample was clustered to assess the presence of any obvious outliers. Then, the optimal soft threshold for adjacency computation was determined, and hierarchical clustering and dynamic tree cut function were used to detect modules. Next, gene significance (GS) and module membership (MM) were calculated to relate modules to clinical traits. Finally, the corresponding module gene information was extracted and the network of eigengenes was visualized. The minimum module size was set to 50, the mergeCutHeight was set to 0.3 and all other parameters were default.

### Bioinformatics and Statistical Analysis

Principal component analysis (PCA) and unsupervised hierarchical clustering analysis were carried out. Interexperiment correlations were calculated by Spearman’s correlation coefficients. The UniProt Gene Ontology Annotation database (http://www.ebi.ac.uk/GOA) was used to annotate the GO terms assigned to the proteins, which were classified as cellular components, molecular functions, kinase, and subcellular location. Functional enrichment analysis was performed using Metascape (https://metascape.org/) to infer dysregulated GO biological processes, KEGG pathways, and Reactome gene sets (18). The background for all enrichment analyses was set to the detected proteome. For all differential expression analysis, a pseudo value of 0.1 was added to buffer against low expression and avoid division by zero before calculating fold-changes and underwent a Benjamini-Hochberg correction (adjusted *p* values). The boxplots of immune organ functions and all heatmaps were displayed based on iFOT values using z-score normalization.

The identification of significantly enriched proteins (D3 *versus* D0) within the MFC tumor involved *t* test incorporating a 10-fold change and an adjusted *p*-value <0.05. Additionally, the Kruskal-Wallis H test was utilized to discern differentially expressed proteins (DEPs) across the three phases (Ph2-Ph4). Proteins exhibiting adjusted *p*-values <0.05 were given priority. Tumor proteins with similar expression patterns were clustered according to the R package Mfuzz (19).

CIBERSORT was used for the immune cell analysis of the tumor protein expression data (20, 21). The 24 tumor samples in the MFC model and 15 tumor samples in Hepa1-6 model data were uploaded to CIBERSORT in RStudio as a mixture file, and CIBERSORT was run with the following options: mouse signature gene file from a literature “Inference of immune cell composition on the expression profiles of mouse tissue” (22), 1000 permutations, and quantile normalization disabled.

For the immune organ analyses, each timepoint (D3, D7, D10, D14, and D21) was individually compared to D0 for differential analysis. The *t* test was performed for proteomic data and proteins with adjusted *p*-value <0.05 and a fold change ≥2 or ≤0.5 were classified as significantly upregulated or downregulated proteins. GPs linked to specific LN/MLN/spleen functions were selected by Metascape analysis on the DEPs.

The criteria used for the selection of highly expressed drug target proteins in tumors were as follows: (1) Kruskal-Wallis H tests were conducted for four tissue types, and the resulting adjusted *p*-value had to be less than 0.05; (2) The average expression level of the protein in the tumor should exceed 1.2 times that of the other three immune organs.

### Xenograft Assay in BALB/c Nude Mice for Target Validation

Male BALB/c nude mice (from Beijing Vital River Laboratory Animal Technology Co, Ltd, Beijing, China), aged 6 weeks, received subcutaneous implantation of small tumor masses derived from the MFC tumor, which was cultivated in the 615 mouse strain and collected on day 61. Following implantation, weekly monitoring of the mice using calipers was performed, and tumor volumes were calculated using the formula (length × width ˆ2)/2. Once the tumors reached a volume of approximately 100 mm^3^, the mice were orally administered trametinib (1 mg/kg), sitravatinib (20 mg/kg), or a combination therapy daily. Monitoring for tumor growth and overall health occurred every 2 days. Upon reaching a tumor size of 2000 mm^3^, euthanasia of the mice was performed.

## Result

### A Temporal Proteomic Atlas of Tumor and Immune Organs in the MFC Subcutaneous Tumor Model

**Fig. 1:**
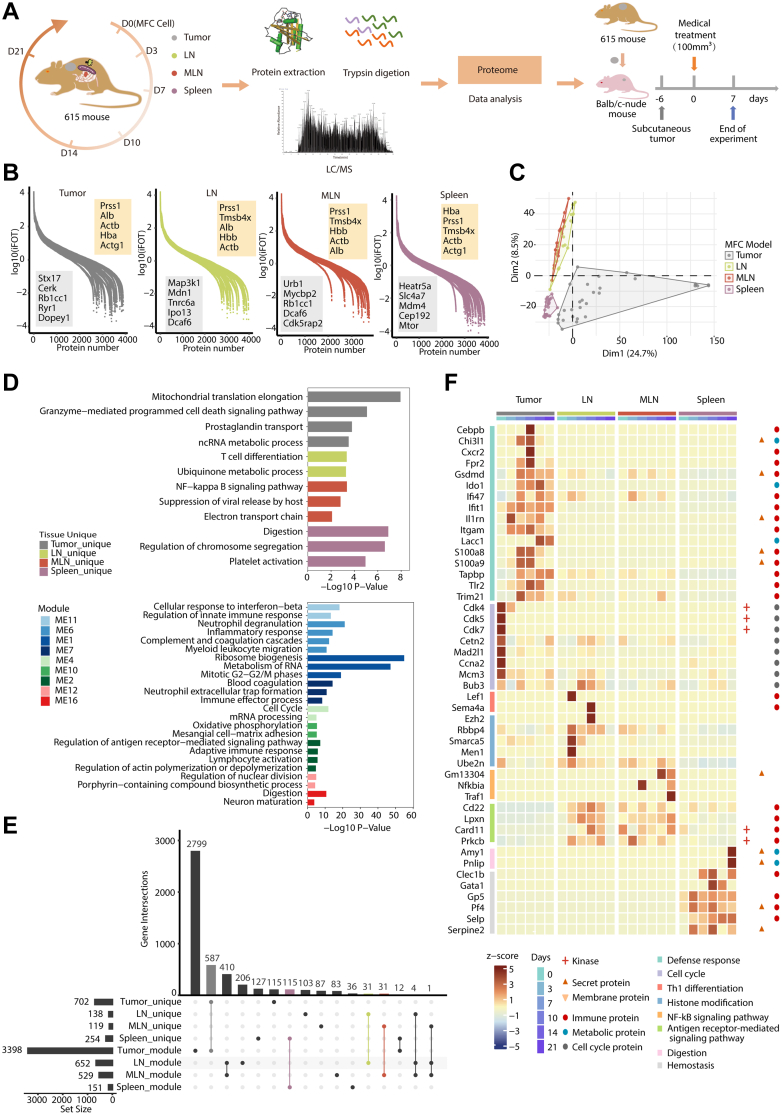
**A summary of dynamic proteomic analysis of the developing tumor and immune organs from the MFC subcutaneous mouse model.***A*, study design and illustration of sample collection, preparation, LC-MS/MS, and experiment validation. LN, draining lymph node; MLN, mesenteric lymph node. *B*, dynamic ranges of protein abundances identified in mouse tumor and three immune organs. The highest-abundance proteins are visually highlighted in *yellow boxes*, while the lowest-abundance proteins are represented in *grey boxes*. *C*, principal component analysis (PCA) of the proteome data on the four tissue samples. *D*, pathway enrichment analysis of tissue-unique proteins and tissue module proteins. *E*, upset plot of the intersection of proteins in tissue-unique and different tissue module proteins. *F*, heatmap of the tissue-specific proteins contained by the proteomic expression alone (determined by Venn diagrams) and the corresponding modules (determined by WGCNA). Tissue-unique proteins, determined by Venn diagrams with tissue expression alone; Tissue module proteins, determined by WGCNA with tissue corresponding modules.

We conducted a temporal proteomic analysis on mouse subcutaneous tumors and three immune organs collected on day 0, 3, 7, 10, 14 and 21 after MFC cell inoculation. Proteomic changes in the immune organs, such as draining lymph node (LN), mesenteric lymph node (MLN), and spleen, could reflect the host immune response to MFC tumor implantation. We performed the whole proteomic profiling of fresh tissue samples using liquid chromatography-tandem mass spectrometry (LC-MS/MS) (Fig. 1*A*). The instrument was operated in the DDA mode; a label-free, intensity-based absolute quantification (iBAQ) approach was used to calculate relative protein abundance (23, 24), and further normalized into iFOT (intensity-based fraction of total multiplied by 10^5^). In all, we identified a total of 12,160 gene products (GPs) at a 1% peptide false discovery rate (FDR; supplemental Fig. S1*A* and supplemental Table S1), of which 7950 GPs were identified by at least one unique peptide and two strict peptides (ion score ≥20), or three strict peptides, or one unique peptide with ion score equal or greater than 40 (supplemental Table S2). We further filtered for higher confidence IDs, requiring that a GP be identified in at least two of the three replicates at one time point, resulting in 6401 high confidence GPs (including 5202 GPs from tumor, 4997 GPs from LN, 4792 GPs from MLN, and 4902 GPs from spleen) (supplemental Fig. S1*B* and supplemental Table S1). Among the 6401 GPs, 3757 were detected in all tissues, 702 were detected in tumor samples only, 138 in LN, 119 in MLN, and 254 in spleen only (supplemental Table S1*C* and supplemental Table S4). The relative abundance (iFOT) of the GPs spanned nearly eight orders of magnitude (Fig. 1*B*). All experiments were highly reproducible as shown by the high correlation coefficients (R = 0.76–0.96) between replicates (supplemental Table S1*D*).

The tumor was clearly separated from the three immune organs by three clustering methods: the PCA, hierarchical clustering (HC), and intersample correlation (Fig. 1*C* and supplemental Table S1, *D* and *E*). Meanwhile, the LN and MLN exhibited a tendency of group together in all three analyses. As shown in supplemental Table S1*F*, subcellular localizations of tumor proteins were annotated as the nucleus, plasma membrane, cytoskeleton, extracellular matrix (ECM), or in extracellular space, suggesting that the MFC proteome also included tumor microenvironment.

To assess the covariance and correlation of proteins and understand the difference across different tissues, we used the Weighted Gene Co-expression Network Analysis algorithm (WGCNA) to identify features of high correlation modules in the corresponding tissues (supplemental Table S1*G* and supplemental Table S5). The ME11, ME6, ME1, and ME7 modules (tumor) were enriched with proteins in the immune system and metabolism process, including cellular response to interferon-beta, neutrophil degranulation, inflammatory response, ribosome biogenesis and mitotic G2-G2/M phases (Fig. 1*D*), suggesting strong immune infiltration in the tumor. Proteins in the ME4 (LN) module were mainly enriched in the cell cycle, and those in the ME10 (MLN) were enriched in oxidative phosphorylation, whereas those in the ME2 module (LN and MLN shared) were enriched in the regulation of antigen receptor-meditated signaling pathway and adaptive immune response, consistent with their tissue-specific functions. Interestingly, the ME12 module (spleen) showed enrichment in the regulation of nuclear division and digestion (Fig. 1*D*), such as the Amylases family and PNLIP (supplemental Table S1*H*). Amylases are secreted proteins that hydrolyze 1,4-alpha-glucoside bonds in oligosaccharides and polysaccharides, thus catalyzing the first step in digestion of dietary starch and glycogen (25). PNLIP is secreted by the pancreas and hydrolyzes triglycerides in the small intestine, and is essential for the efficient digestion of dietary fats (26). These proteins were detected in the spleen only on D21 (supplemental Table S1*H*). The tissue-specific proteins were determined as the common ones from proteomic expression alone (tissue-unique proteins) and those identified by the corresponding modules (tissue-module proteins) (Fig. 1, *E* and *F*). The pathway enriched by the two methods are shown in Figure 1*D*. Together, our analysis suggests that the malignant proliferation of the tumor elicited the body’s defense response, including LN and MLN-regulated T cell differentiation, NF-kappa B and antigen receptor-mediated signaling pathway to interact with tumor, remodeled metabolism, and enhanced blood coagulation by spleen in response to tumor perturbation (Fig. 1*F*).

### Dynamics Proteomic Changes and Four Distinct Phases of Tumor Growth

**Fig. 2:**
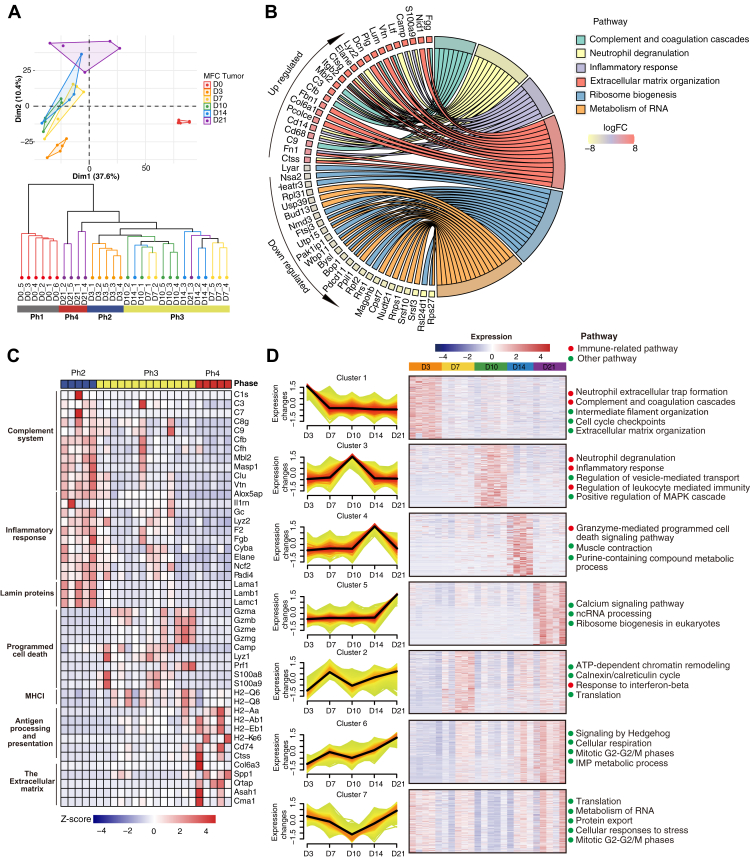
**Four major tumor development phases and relative biological process including representative proteins.***A*, PCA analysis and hierarchical clustering (HC) of temporal tumor proteomic data to separate the whole developmental process into four phases. *B*, the chord plot representing the regulated pathway and significant proteins between tumor D3 and D0. The color gradient from *yellow* to *red* represents down-regulated and up-regulated protein levels, respectively. *C*, a heatmap of selected proteins representing major altered signaling pathways across 24 tumor samples from D3 to D21 in Ph2-4. *D*, *left panel*: Seven protein clusters revealed by co-expression analysis using the fuzzy c-means (FCM) clustering algorithm; *Right panel*: representative pathways of each cluster.

Murine syngeneic tumor models are important for understanding the interaction between tumor and microenvironment and for validating novel targeted therapy (27). Although the whole tumor grows gradually, phases that separate critical events may exist that indicate profound changes during the tumor growth. To investigate these events at the protein expression level, we first carried out PCA and HC to identify discernable boundaries that demarcate the key stages with distinct features. The six timepoints could be grouped into four distinct phases: Ph1 (D0), Ph2 (D3), Ph3 (D7–D14), and Ph4 (D21) (Fig. 2*A*). As expected, there was a profound change in D3 when the MFC cells took root and grew into tumor. Differential expression analysis between D3 and D0 identified 364 upregulated and 2192 down-regulated proteins (*t* test adjusted *p*-value <0.05 and 2-fold change). Among them, 227 were up-regulated and 469 were downregulated by 10-fold (Fig. 2*B* and supplemental Table S6). Immune response and ECM proteins were up-regulated, whereas malignant proliferation of tumor cells, including mRNA metabolism and ribosome biogenesis proteins, were down-regulated (Fig. 2*B*).

We identified representative proteins in each phase after D3 and determined their biological functions (Fig. 2*C*). Ph2 highly expressed proteins in all three branches of the complement system (classical pathway, mannose-binding lectin pathway, and alternative pathway), which triggers membrane attack, phagocytosis and inflammation by attracting macrophages and neutrophils. For example, ELANE (Neutrophil elastase), is released catalytically from neutrophils to kill many cancer cell types (28). LYZ2 (Lysozyme) has primarily a bacteriolytic function. Importantly, Ph2 also highly expressed the lamins in the ECM, which activate various signal transduction pathways, promote tumor cell attachment and proliferation, and induce invasion (29). Ph3 appears to be the phase during which the tumor was subjected to attack by the host immune systems. Proteases such as granzymes (GZMA, GZMB, GZME, and GZMG) in the cytosolic granules of cytotoxic T-cells and NK-cells, which activates caspase-independent pyroptosis when delivered into the target cell through the immunological synapse (30), were detected in high abundance. Meanwhile, MHC I (Major Histocompatibility Complex, Class I, A) molecules that play a central role in the immune system by presenting peptides were high expressed in Ph3. MHC-I-restricted neoantigens are important targets of tumor-specific CD8^+^ cytotoxic T lymphocytes (CTL). The last phase Ph4 involves antigen processing and presentation and the build-up of the ECM. Tumors leverage ECM remodeling to create a microenvironment that promotes tumourigenesis and metastasis (31). The antigen processing and MHC II expression may play an important role in anti-tumor response as these proteins were highly expressed in Ph4.

Next, we analyzed the dynamic changes in MFC tumor proteins (DataSet 3) using Fuzzy C-Means Clustering (FCM), a soft clustering method. Since the protein expression profile of the MFC cells (D0) was so profoundly different from those of implanted tumors, we excluded the MFC cell data points on D0, such that a better resolution during the tumor growth could be achieved. We identified seven protein expression patterns and determined their functional enrichment (Fig. 2*D* and supplemental Table S7). Proteins in Cluster 1 showed a sharp decline in abundance after D3, and functional enrichment showed that they are involved in neutrophil extracellular trap formation, complement activation, and cell cycle. Proteins in Cluster 2 showed a significant rise on D7 followed by a decline on D10 and then an increase until D21. These proteins function mainly in ATP-dependent chromatin remodeling, response to interferon-beta, and translation. The Cluster 3 proteins reached a peak on D10 followed by a steady decline; they were enriched in the activated immune system. The Cluster 4 proteins that peaked on D14 were enriched in granzyme-mediated programmed cell death signaling pathway and purine-containing compound metabolic process. Two clusters (Cluster 5 and Cluster 6) that showed the highest abundance on D21 were enriched mainly in nucleotide metabolism, cellular respiration and signal transduction. Cluster 7 decreased sharply from D3 and then increased from D10, which showed pathways in the metabolism of RNA, cellular response to stress, and cell cycle. Together these results suggest that starting from D10, the tumor had overcome the inhibition by the immune system and resumed growth as evidenced by the increase in cell cycle, metabolism of RNA, and ribonucleoprotein complex biogenesis.

### Dynamic Proteomic Changes in LNs and Spleen and Their Functional Implications

LNs serve as education centers for the immune system where antigen-presenting cells (APCs) prime T cells prior to their egress and elimination of cells harboring those antigens (32). Draining lymph nodes are close to the tumor, whereas mesenteric lymph nodes are located in the small intestine. It has also been observed that immune cell states are dynamically altered across immune organs with tumor growth (10). Next, we examined proteomic changes in the immune organs during tumor implantation and growth.

**Fig. 3:**
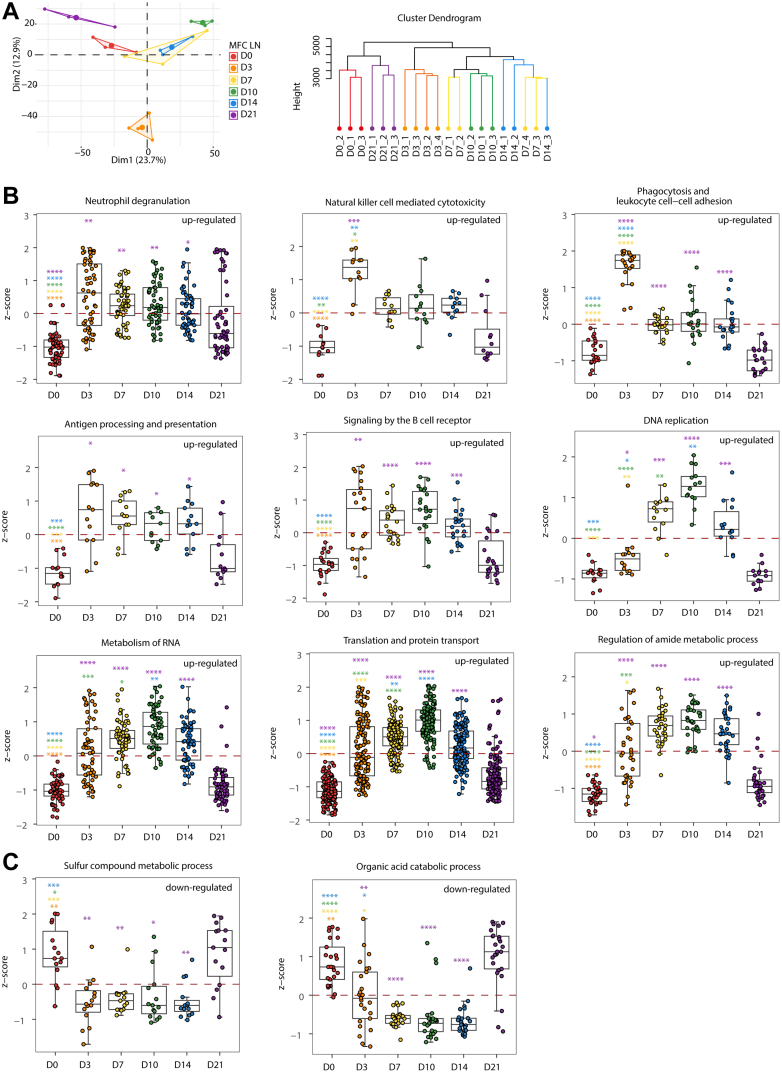
**Temporal expression patterns of LN function-associated proteins across MFC tumor development.***A*, PCA and hierarchical clustering of the temporal LN proteomics data in MFC model. *B*, the upregulated LN functions during tumor development were depicted through the z-score derived from the up-proteins, which were classified based on their differential expression in LN D3/7/10/14/21 compared to D0, respectively. *C*, the downregulated LN functions during tumor development were depicted through the z-score derived from the down-proteins. ∗*p* < 0.05; ∗∗*p* < 0.01; ∗∗∗*p* < 0.001; ∗∗∗∗*p* < 0.0001; *t* test. The color of each *asterisk* at respective time points denotes the comparison between the current time point and the time point associated with the same color of *asterisk*.

Using both HC and PCA, the LN datapoints were classified into three clusters with minor difference from the tumor classification (Fig. 3*A*). Notably, the D3 datapoint was separated furthest from D0 and the rest of the datapoints. It appeared that tumor inoculation gave rise to the greatest perturbation for the LN homeostasis, but it gradually returned to its original state. We next identified proteins that were differentially expressed between LNs in tumor-bearing and unperturbed mice (D0). Among the 4998 proteins (LN in DataSet 3) that were quantified, 324, 343, 696, 219, and 97 proteins were significantly up-regulated on LN D3/7/10/14/24, respectively, compared to LN D0 (supplemental Table S8). Proteins altered in perturbed LNs were enriched in the metabolism of DNA replication, gene expression-related pathways, and immune-related pathways from the common knowledge databases (Gene Ontology, KEGG, and Reactome). We then mapped the temporal patterns of the significantly altered biological processes along the tumor growth time scale (Fig. 3*B*). The increase in innate immune-related processes, such as neutrophil degranulation, cytotoxicity, phagocytosis, and leukocyte cell-cell adhesion occurred rapidly on D3, suggesting that LN mounted an acute defense response to tumor cell implantation. Adaptive immunity (antigen presentation and B cell receptor signaling) was stimulated on D3 after tumor implantation and lasted until D14, implying the immune stress was limited and eventually entered the equilibrium stage. On the other hand, increase in the metabolism of RNA, translation, DNA replication, and amide metabolism was relatively moderate but persistent, reaching peak responses on D10 (Fig. 3*B*), indicating that LN adapted the change by increased proliferation. In contrast, sulfur compound metabolic process and organic acid catabolic process were decreased upon MFC cell implantation and remained at low levels through D14 (Fig. 3*C*).

Both PCA and HC of MLN showed that most significant change occurred on D3. While the state of MLN continued to change on D7, it remained relatively stable until D21 (supplemental Fig. S2*A*). Detailed pathway enrichment showed that upon tumor implantation, intracellular protein transport, ATP metabolism and neutrophil degranulation were increased (supplemental Fig. S2*B* and supplemental Table S9), metabolism of RNA, regulation of leukocyte cell-cell adhesion were decreased (supplemental Fig. S2*C* and supplemental Table S9). The only process that returned to the original state after an initial drastic increase on D3 was translational initiation complex formation. This is in stark contrast to the states of draining LN, where the majority of the processes returned to their original states.

**Fig. 4:**
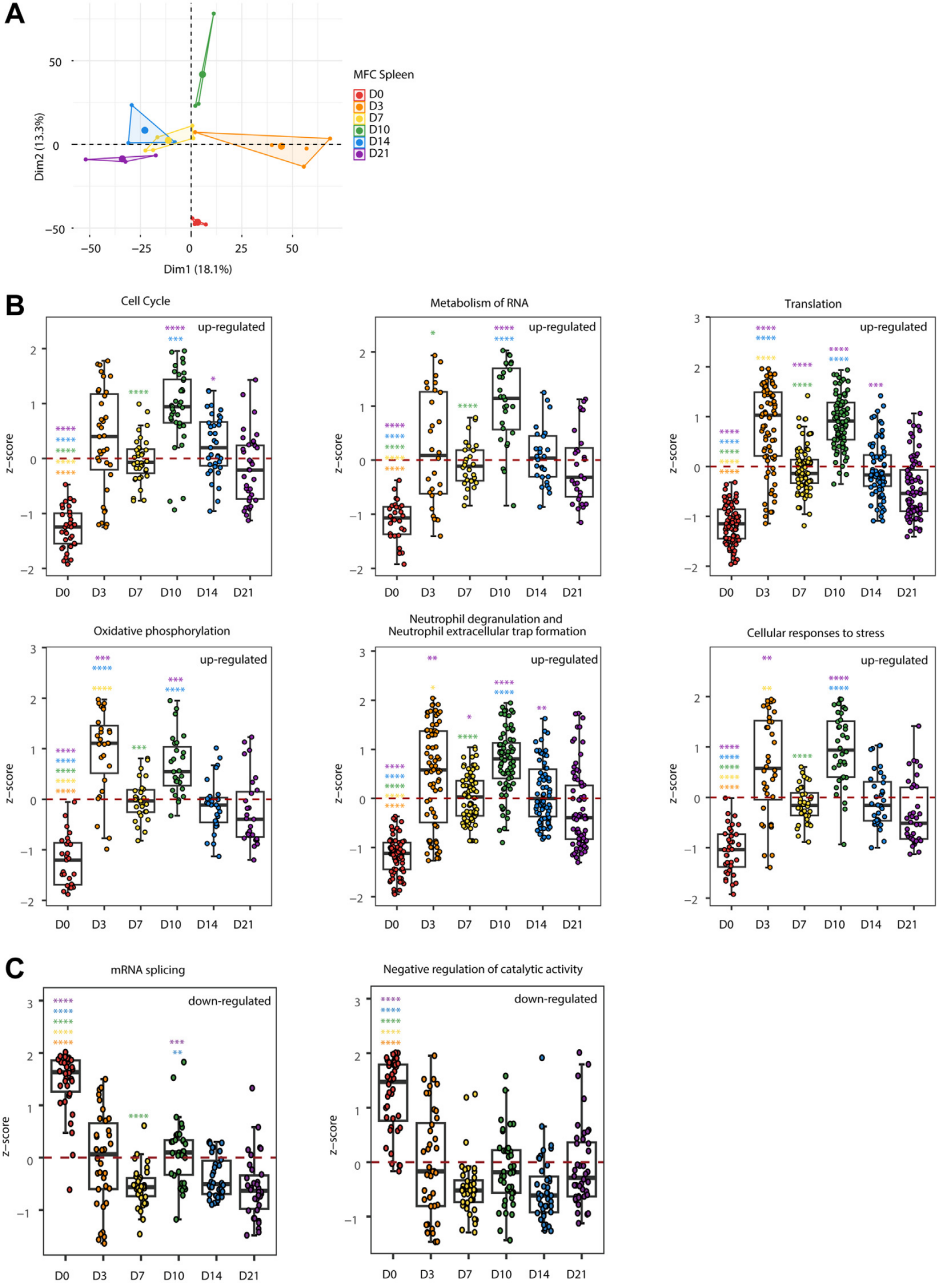
**Temporal expression patterns of spleen function-associated proteins across MFC tumor development.***A*, PCA of the temporal spleen proteomics data in the MFC model. *B*, the upregulated spleen functions during tumor development were depicted through the z-score derived from the up-proteins, which were classified based on their differential expression in spleen D3/7/10/14/21 compared to D0, respectively. *C*, the downregulated spleen functions during tumor development were depicted through the z-score derived from the down-proteins. ∗*p* < 0.05; ∗∗*p* < 0.01; ∗∗∗*p* < 0.001; ∗∗∗∗*p* < 0.0001; *t* test. The color of each *asterisk* at respective time points denotes the comparison between the current time point and the time point associated with the same color of *asterisk*.

The spleen is a part of immune surveillance where lymphocytes are produced. To investigate the role of the spleen in tumor-host interaction and tumor progression (33), we performed a similar proteomic analysis on the MFC spleen (Fig. 4*A*). Unlike the trend observed in LN, all six upregulated processes, including cell cycle, metabolism of RNA, translation, oxidative phosphorylation, neutrophil degranulation, and NET formation, and cellular response to stress, appeared to exhibit two phases: an acute response on D3 followed by a decrease, then a second response that peaked on D10 (Fig. 4*B* and supplemental Table S10). Similar to LN, these processes also returned to their original states by D21. In contrast, the downregulated processes, including mRNA splicing and negative regulation of catalytic activities, didn’t return to the original states (Fig. 4*C* and supplemental Table S10). A consistent valley on D7 and D14 in both up and down processes suggest that the spleen had the ability to continuously restore homeostasis.

### Profiling Tumor Infiltrating Immune Cells With CIBERSORT

**Fig. 5:**
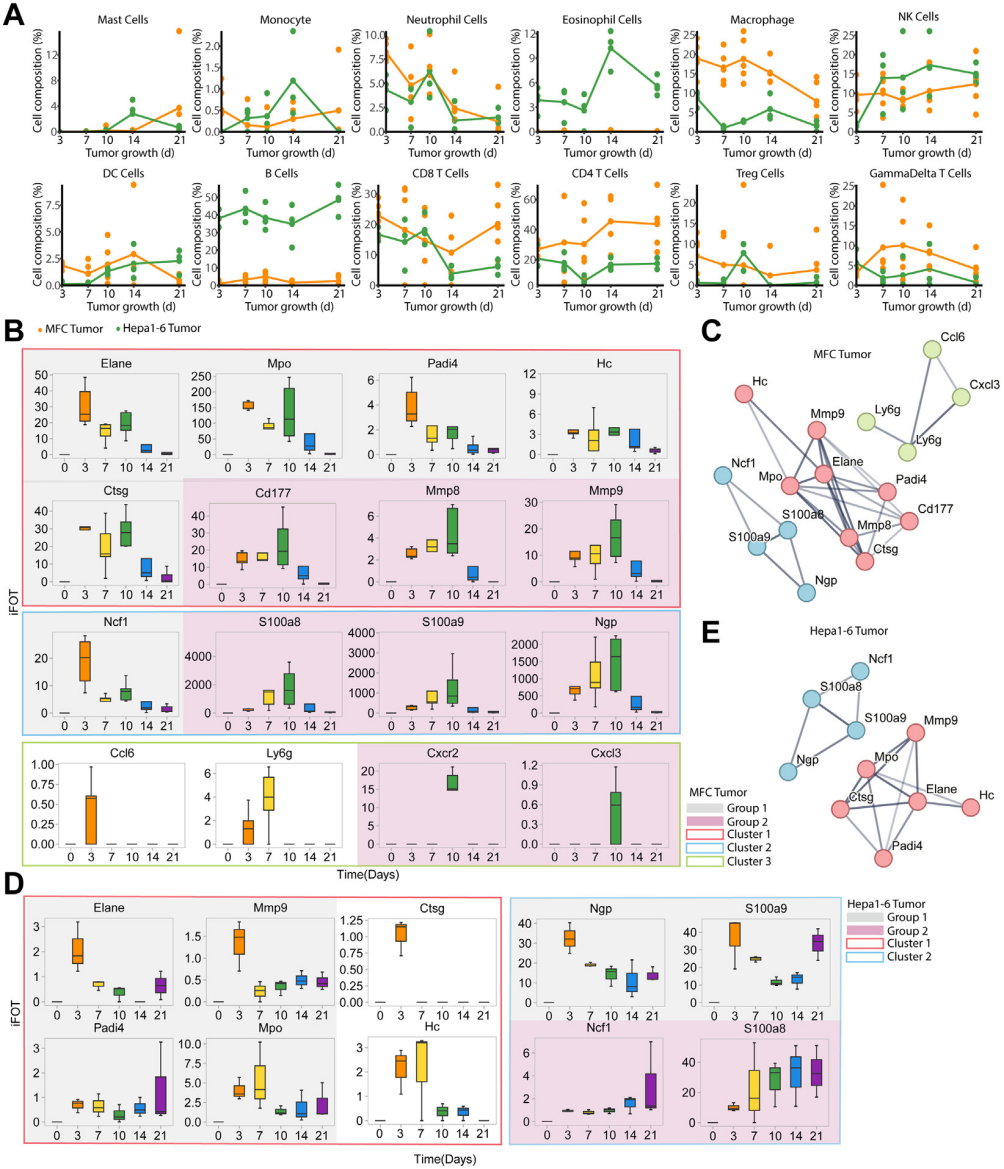
**The immune cell infiltration analysis with tumor development.***A*, curves of mean cell frequencies across time from 12 immune cell types by CIBERSORT in tumor samples. *Orange*, MFC tumor; *Green*, Hepa1-6 tumor. *B*, sixteen neutrophil-related proteins were classified in three clusters by k-means in String and into two distinct groups based on their expression patterns in MFC tumors. The different *solid line boxes* represent distinct clusters of proteins, while varying colored shades denote different groups. *C*, the PPI network of 16 neutrophil-related proteins in MFC tumor was classified in 3 clusters. *D*, ten neutrophil-related proteins were classified in 2 clusters by k-means in String and into two distinct groups based on their expression patterns in Hepa1-6 tumor. *E*, PPI network of ten neutrophil-related proteins in Hepa1-6 tumor was classified into two clusters.

In progressing cancers, neither the tumor nor the TME is static. Reciprocal interactions between tumor and associated immune and stromal cell types evolve as the tumors grow, thus allowing for modulation of both tumor cell intrinsic and extrinsic processes (34). Previous studies have shown that different tumor models have unique systemic immunological landscape and response to tumor development. To assess immune cell infiltration, we applied CIBERSORT, a deconvolution algorithm with pre-defined immune cell RNA expression signatures, to the temporal tumor protein expression data of MFC. The same analysis was also conducted on Hepa1-6, a hepatocellular carcinoma mouse model, which had been previously examined for dynamic proteomic profiles (35). Among the 25 immune cell types available in the CIBERSORT database, 12 were identified in the two tumor models (Fig. 5*A* and supplemental Table S11). CD4^+^ T cells were the most abundant population in MFC tumor model, representing 35.7% of the leucocytes, followed by CD8^+^ T, Macrophage, and NK cells; in contrast, B cells were the most abundant population in Hepa1-6 tumor model, representing 31.7% of the leukocytes, followed by CD8^+^ T, CD4^+^ T, and NK cells. In both MFC and Hepa1-6 tumor models, macrophage and CD8^+^ T cells showed a decreased tendency between D3 and D21(Fig. 5*A*), suggesting a weakened ability of cell killing by T cells on D21. These results suggest that the anti-tumor adaptive immunity is mainly achieved by T cells in the MFC tumor model and both B and T cells are employed in the Hepa1-6 model.

Neutrophils showed a consistent trend in MFC and Hepa1-6 tumor models. They peaked on D3 and D10 and then gradually decreased to almost undetectable levels (Fig. 5*A*). String Database classified the 16 neutrophil proteins into 3 clusters (Fig. 5, *B* and *C*): The cluster one proteins (ELANE, MPO, PADI4, CD177, CSTG, HC, MMP8, and MMP9) were mainly neutrophil proteases involved in degrading ingested host pathogens and neutrophil extracellular traps (NETs). The cluster 2 proteins (NCF1, NGP, S100A8, and S100A9) are related to promoting inflammation and activation of the neutrophilic NADPH-oxidase. The cluster 3 proteins (Ly6G, CXCR2, CCL6, and CXCL3) mediate neutrophil migration to sites of inflammation. Detailed examination of the expression of the 16 neutrophil-enriched proteins in MFC showed that they can be categorized into two groups: Group 1 proteins (ELANE, MPO, PADI4, NCF1, HC, and CSTG) exhibited a sharp peak on D3, a slight decrease on D7, followed by a second peak on D10. Group 2 proteins, including CD177, MMP8, MMP9, S100A8, S100A9, NGP, CXCR2, and CXCL3, showed only one peak on D10 (Fig. 5*B*). These results suggest that the group 1 proteins amounted an acute and transient response to the implantation consistent to events occurred in Ph2 in tumor (Fig. 2*C*), whereas both group 1 and group 2 proteins were involved in the adapted response occurred in Ph3 in the tumor (Fig. 2*C*).

The response pattern in Hepa1-6 also exhibited two patterns that were different from those of MFC (Fig. 5, *B* and *D*). Group 1 proteins, including cluster 1 components (ELANE, MMP9, PADI4, and MPO, and cluster 2 components NGP, S100A9) showed a transient increase on D3 followed by a steady decrease until D14, and then started to increase again on D21. Group 2 proteins, including NCF1 and S100A8, two components of cluster 2, showed a continuous increase (Fig. 5, *D* and *E*). Calprotectin (S100A8/A9), a heterodimer of the two calcium-binding proteins S100A8 and S100A9, was originally discovered as an immunogenic protein expressed, secreted by neutrophils and often co-expressed (36). However, S100A8 and S100A9 in the Hepa1-6 model showed expression patterns different from the MFC model (Fig. 5, *B* and *D*), indicating that they also had different functions in tumorigenesis and development. These results suggest that although neutrophil recruitment to tumors exhibited similar patterns, their protein components may execute distinct functions in a tumor model-specific manner.

### Identification of Potential Drug Targets for the Tumor Treatment

**Fig. 6:**
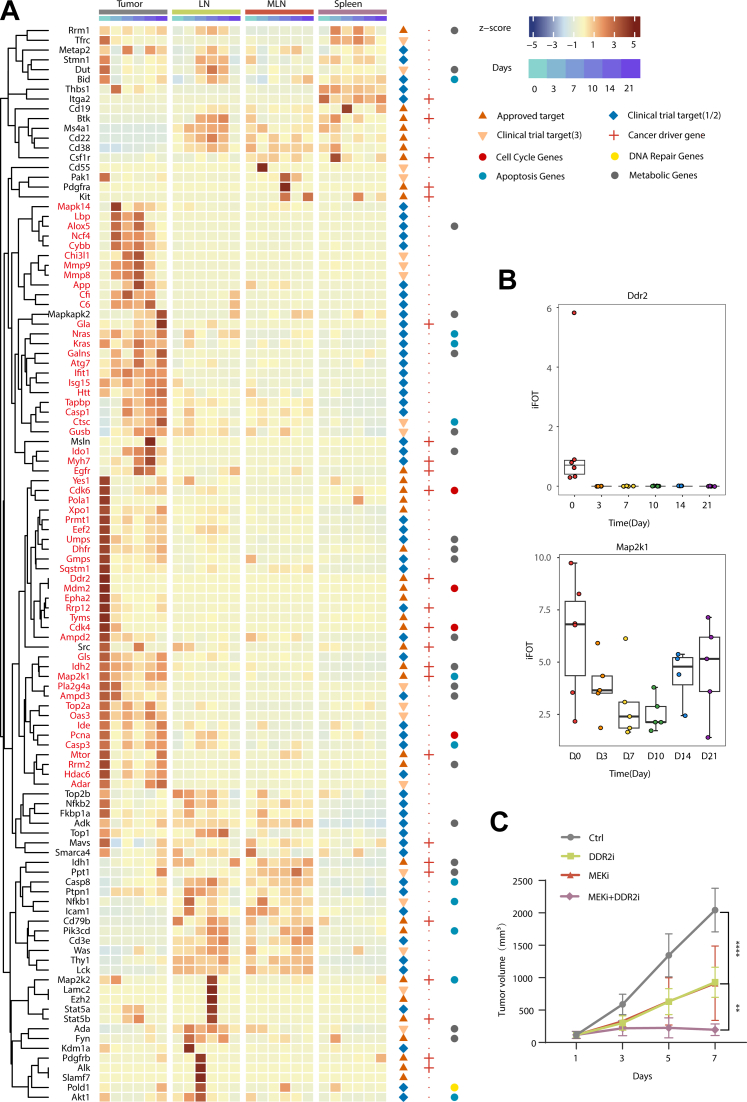
**Potential druggable proteins for MFC model.***A*, 110 drug targets were identified according to the GeneBank Database. Of them, 57 proteins were highly expressed in tumors. *B*, temporal expression levels of DDR2 and MAP2K1 by the protein measurements. The *line* and *box* represent the median and upper and lower quartiles, respectively. *C*, tumor growth curve of MFC xenografts (n = 5; Mean ± SD) administered drugs as single agents or in combination (MAP2K1-targeting trametinib or DDR2-targeting sitravatinib). ∗∗*p* < 0.01; ∗∗∗∗*p* < 0.0001; two-way ANOVA.

To explore potential therapeutic targets for cancer treatment, we cross-referenced the 6401 proteins to drug targets in the DrugBank (37) and identified 110 proteins as potential drug targets (Fig. 6*A* and supplemental Table S12). Among them, 57 proteins were expressed higher in tumor than other tissues, which could be targeted for treatment. Of the 57 proteins, 26.3% (15/57) of the proteins are approved targets for treating solid tumors, 15.8% (9/57) in phase III clinical trials and 57.9% in phase I/II clinical trials. Functional annotation of the 57 proteins indicated that 17.5% (10/57) of them are cancer driver gene, 7% (4/57) are involved in the cell cycle, 8.8% (5/57) are apoptosis genes, and 21% (12/57) are metabolic genes (Fig. 6*A*).

We selected two representative proteins, MAP2K1 and DDR2, for a prove-of-concept validation study (Fig. 6*B*). The mitogen-activated protein kinase (MAPK) pathway is a highly conserved signal transduction pathway in all eukaryotic cells that regulates a variety of normal cellular functions, including cell proliferation, differentiation, survival, and apoptosis (38). MAP2K1 is considered a central component in the MAPK signaling pathway (39). DDR2 (Discoidin Domain Receptor Tyrosine Kinase 2) is thought to mediate tumor cell-collagen interactions at various stages of cancer progression, where they initiate signal transduction pathways that regulate epithelial-to-mesenchymal transition (EMT), cell proliferation and survival, and metastatic dissemination (40). Moreover, the mouse forestomach tumor from which the MFC cell line was derived had developed spontaneous lung metastasis (13), making DDR2 a relevant target.

To examine the effect of MAP2K1 and DDR2 inhibition on tumor growth, mice bearing MFC tumors were injected with MAP2K1-targeting trametinib or DDR2-targeting sitravatinib (supplemental Fig. S3). Tumor growth was monitored for 7 days. The growth curves indicated that trametinib or sitravatinib treatment alone led to a 49.97% and 52.97% reduction on D7, respectively, in subcutaneous tumor growth model (Fig. 6*C*). Importantly, a combined therapy with both trametinib and sitravatinib treatment led to a more efficient effect, resulting a 90.92% reduction on D7 (Fig. 6*C*). Together these results demonstrated the feasibility of using proteomics to identify treatment targets and its potential application in personalized precision therapy.

## Discussion

Compared to PDX model, immunocompetent mouse models are important tools for understanding the role of the immune system in tumor biology. Several studies have shown that a systemic anti-tumor immune response is essential for immunotherapeutic efficacy (41). However, a comprehensive investigation on how cancer development affects the systemic immune state is lacking (10).

The current study presented an overview of dynamic proteomic changes of the mouse MFC cell-derived subcutaneous tumor and three host immune organs during implantation. The CIBERSORT-based deconvolution algorithm was used to examine dynamic changes in the immune cell population. Our bioinformatic analysis suggests that the tumor implantation and development period may be classified into distinct phases, and the key events can be recapitulated in the proteomic changes in immune organs.

Our data showed that Ph2 (D0 to D3) is a period for immune system recognition and activation, and Ph3 (D7 to D14) is associated with cell cycle and adaptive immunity, and the last phase, Ph4, is a phase highlighted by increased RNA metabolism and immune evasion. These results provide proteomic evidence to support a previous hypothesis of host “immunoediting” processes, which include elimination, equilibrium, and escape (42). The expression of tumor-specific antigens underlies cancer immunoediting, and the expression of MHC-I in MFC tumor model is consistent with this process. Meanwhile, MHC-II is critical for antigen presentation to CD4^+^ T cells, which play an important role in supporting CD8^+^ T cell activation, generation of memory T cells, and their effective response to ICI (43). However, MHC-II was high in “escape” phase, which may create an immunosuppressive microenvironment by activating CD4^+^ T cells.

In the MFC tumor model, CD4^+^ T cells dominated the immune landscape, while B cells were most prevalent in the Hepa1-6 model. Both models showed a decline in macrophage and CD8^+^ T cell populations over time, hinting at a reduced T cell-mediated cell-killing function by day 21. Neutrophil levels peaked early but diminished by day 21 in both models. Proteins from neutrophils formed distinct clusters linked to defense response, inflammation, and migration. The expression of these proteins differed between models, indicating model-specific roles in tumor progression and immune response. Together, these results suggest that dynamic changes of infiltrating immune cells and the functions of systemic immunity organs of MFC and Hepa1-6 syngeneic models were different. It appears that the MFC model is more immunosuppressive, either because the MFC cancer cells are of lower immunogenicity or the host immune system of the 615 mice is more immune suppressive. However, a caveat of applying RNA-based CIBERSORT is the poor correlation between RNA and protein expression, so data should be interpreted with caution.

We have also demonstrated the potential of proteomic-guided precision cancer therapy. We showed that, based on the differential high expression, inhibition of either MAP2K1 or DDR2 reduced tumor growth, and a combination therapy almost completely suppressed tumor growth. Interestingly, the expression of DDR2 in D0 tumor samples was the highest, indicating that early drug treatment may be more effective in slowing down the tumor growth. However, before considering these selected targets as viable candidates for tumor targeting, it will be imperative to thoroughly evaluate their expression profiles across a spectrum of tissues and cell types.

Other potential targets, such as XPO1 may be an attractive pan-cancer target, as it is frequently overexpressed and/or mutated in multiple human cancers and functions as an oncogenic driver (44). XPO1 is an export receptor responsible for the nuclear-cytoplasmic transport of hundreds of proteins and multiple RNA species. XPO1 inhibitor has been approved in treating relapsed and refractory multiple myeloma and relapsed diffuse large B-cell lymphoma, and has the potential for treating stomach cancer. Another good candidate is ADAR. It is the primary enzyme responsible for editing the inverted repeats that form long dsRNAs (45). Recent work reveals that elevated ADAR1 activity in some cancers can efficiently neutralize the immunogenicity of the endogenous dsRNAs, keeping the immune response in check (46, 47, 48). Certain types of tumor cells have a unique vulnerability to ADAR1 loss and deleting ADAR1 could sensitize tumors to immunotherapy (49, 50). ATG7 encodes an E1-like activating enzyme that is essential for autophagy and cytoplasmic to vacuole transport. ATG7 deficiency causes p53 activation, accumulation of defective mitochondria, proliferative defects, reduced tumor burden, conversion of adenomas and adenocarcinomas to oncocytomas, and increased mouse survival (51, 52). It has the potential to develop an ATG7-based specific therapeutic strategy for the treatment of GC and EC. HDAC6, unlike other HDACs, is primarily cytoplasmic in localization and regulates the function of non-histone cytoplasmic proteins like α-tubulin, Hsp90, cortactin, and many more (53). HDAC6 is overexpressed in a variety of tumors including primary acute myeloid leukemia (AML) blasts (54), colon cancer (55), and so on, and plays important roles in various processes, such as enhanced cellular proliferation, cancer cell migration and invasion (56, 57). Also selectively targeting HDAC6 over other subtypes maximizes the pharmacological effects and also minimizes the side effects associated with pan-HDAC inhibitors (53).

In summary, our study investigated the temporal proteomic changes on a specific tumor and its macroenvironment, and provides a valuable resource for further in-depth data interrogation for more effective immunotherapy and precision drug targets in EC and GC.

## Data Availability

The proteomics data, the spectral library, and details regarding all identified proteins have been submitted to the ProteomeXchange Consortium (https://www.iprox.cn/). Project ID: IPX0007136001.

## Supplemental Data

This article contains supplemental data.

## Conflict of interest

The authors declare that they have no known competing financial interests or personal relationships that could have appeared to influence the work reported in this paper.

## Supplemental Data

Supplemental Figure S1 **Data quality control and correlation analysis in four tissues.***A*, datasets (Dataset 1–3) with different confident levels by stepwise filtering criteria. *B*, the total number of gene products identified in each timepoint and tissue. *C*, Venn diagrams summary the number of proteins identified commonly or uniquely. *D*, correlation analysis of experiments between six timepoints across four tissues. *E*, hierarchical clustering of the proteomics data from the four tissues in the MFC model. *F*, subcellular distribution of tumor proteins annotated with Gene Ontology. *G*, heatmap of the correlation between the module eigengenes and traits of the tissues by WGCNA. The number on each cell is the correlation coefficient and the number below is the corresponding p value. *H*, dynamics changes of the proteins involved in digestion in spleen.

Supplemental Figure S2 **Temporal expression patterns of MLN function-associated proteins across MFC tumor development.***A*, PCA and hierarchical clustering of the temporal MLN proteomics data in MFC model. *B*, the up-regulated MLN functions during tumor development were depicted through the z-score derived from the up-proteins, which were classified based on their differential expression in MLN D3/7/10/14/21 compared to D0, respectively. *C*, the down-regulated MLN functions during tumor development were depicted through the z-score derived from the down-proteins. ∗*p* < 0.05; ∗∗*p* < 0.01; ∗∗∗*p* < 0.001; ∗∗∗∗*p* < 0.0001; *t* test. The color of each *asterisk* at respective time points denotes the comparison between the current time point and the time point associated with the same color of *asterisk*.

Supplemental Figure S3 **The illustrative diagram of targeted therapy for MFC tumors**.
