# Supplementary figures and images for: Dynamic Proteomic Changes in Tumor and Immune Organs Reveal Systemic Immune Response to Tumor Development
Source: Mol Cell Proteomics. 2024 Mar 28;23(5):100756. doi: 10.1016/j.mcpro.2024.100756 (PMC11060955; doi:10.1016/j.mcpro.2024.100756)

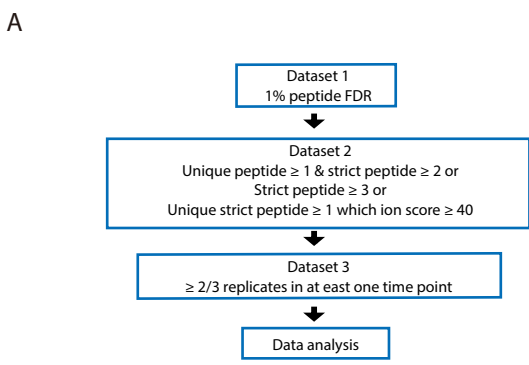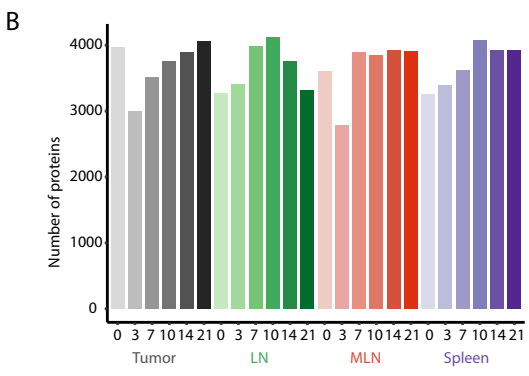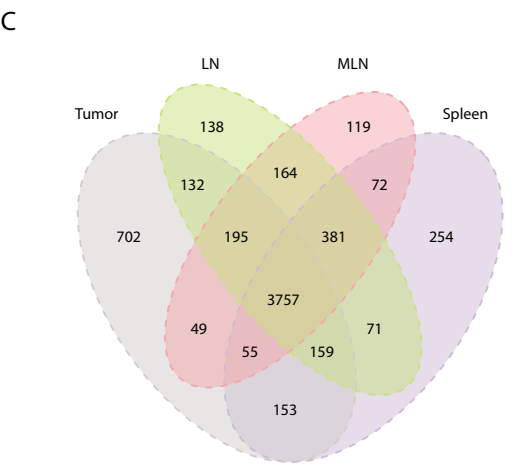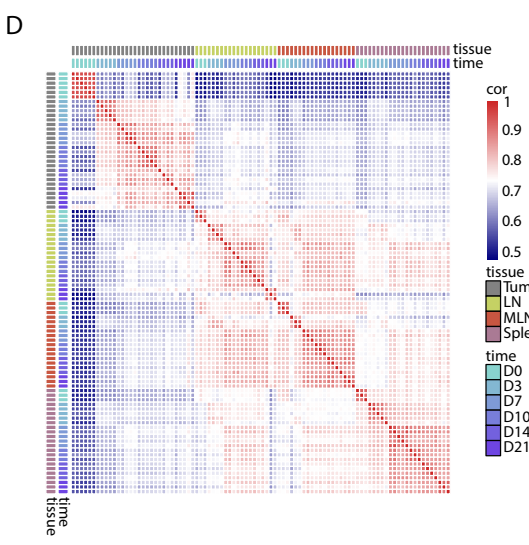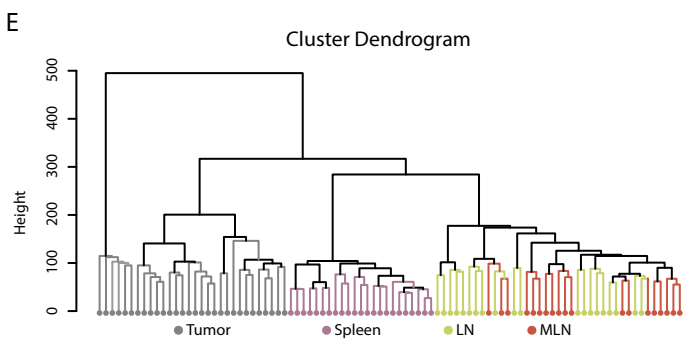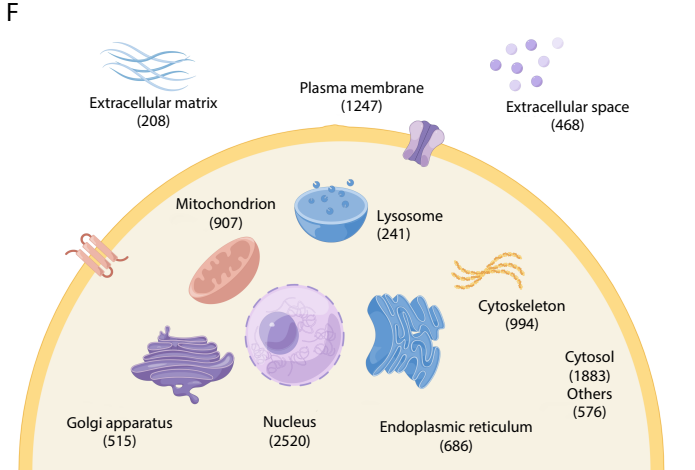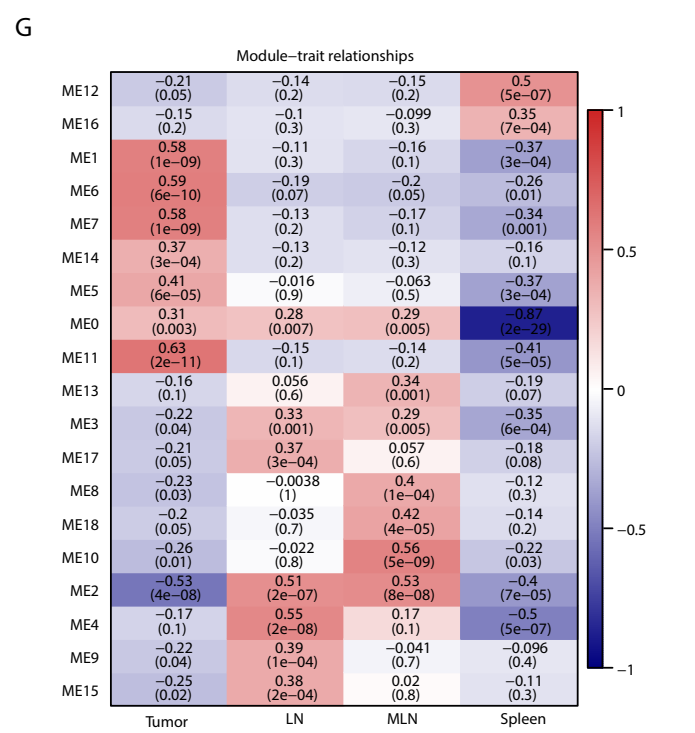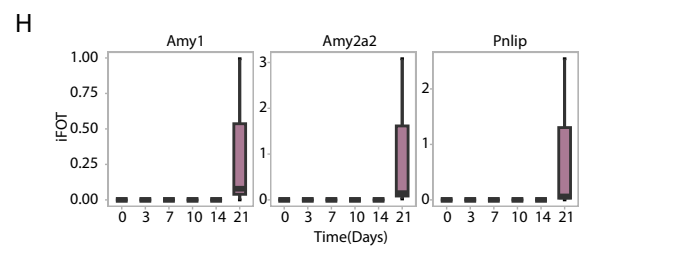

Supplement: Supplemental Figure S1 — Data quality control and correlation analysis in four tissues.A, datasets (Dataset 1–3) with different confident levels by stepwise filtering criteria. B, the total number of gene products identified in each timepoint and tissue. C, Venn diagrams summary the number of proteins identified commonly or uniquely. D, correlation analysis of experiments between six timepoints across four tissues. E, hierarchical clustering of the proteomics data from the four tissues in the MFC model. F, subcellular distribution of tumor proteins annotated with Gene Ontology. G, heatmap of the correlation between the module eigengenes and traits of the tissues by WGCNA. The number on each cell is the correlation coefficient and the number below is the corresponding p value. H, dynamics changes of the proteins involved in digestion in spleen. [file mmc1.pdf]

A

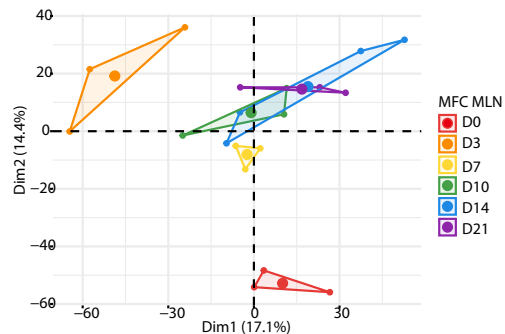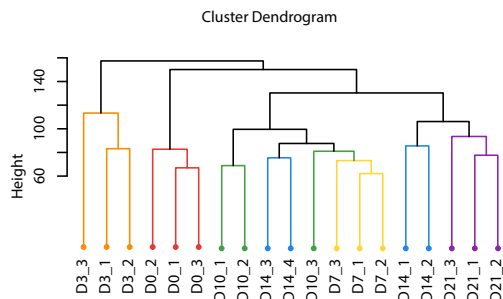

B

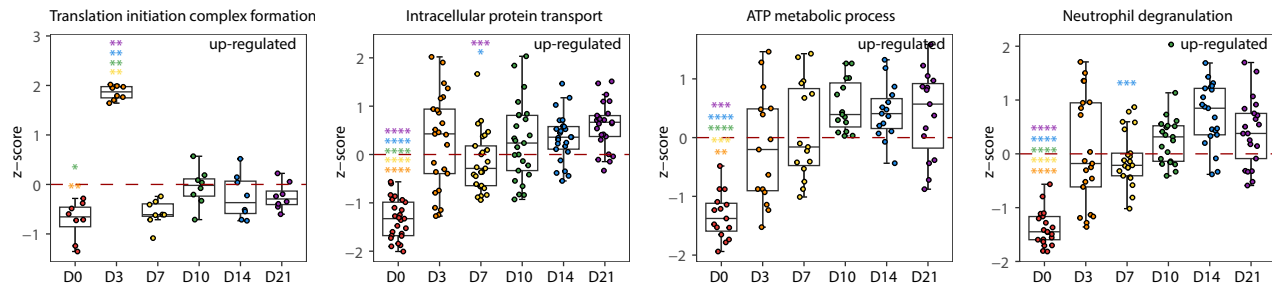

C

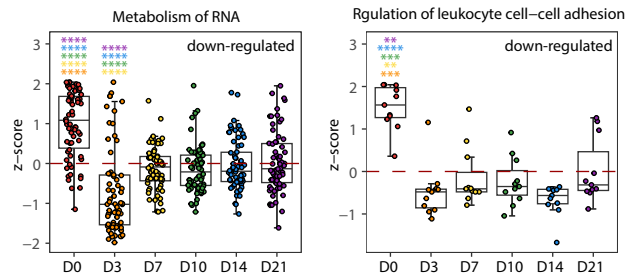

Supplement: Supplemental Figure S2 — Temporal expression patterns of MLN function-associated proteins across MFC tumor development.A, PCA and hierarchical clustering of the temporal MLN proteomics data in MFC model. B, the up-regulated MLN functions during tumor development were depicted through the z-score derived from the up-proteins, which were classified based on their differential expression in MLN D3/7/10/14/21 compared to D0, respectively. C, the down-regulated MLN functions during tumor development were depicted through the z-score derived from the down-proteins. ∗p < 0.05; ∗∗p < 0.01; ∗∗∗p < 0.001; ∗∗∗∗p < 0.0001; t test. The color of each asterisk at respective time points denotes the comparison between the current time point and the time point associated with the same color of asterisk. [file mmc2.pdf]

MFC Cell

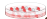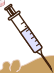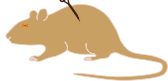

615 mouse

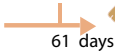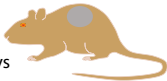

615 mouse

MFC tumor  
mass

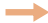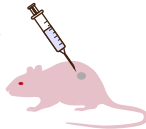

Balb/c-nude  
mouse

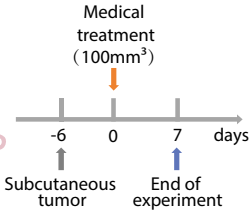

Supplement: Supplemental Figure S3 — The illustrative diagram of targeted therapy for MFC tumors. [file mmc3.pdf]
